# Clinical Significance of Trace Element Zinc in Patients with Chronic Kidney Disease

**DOI:** 10.3390/jcm12041667

**Published:** 2023-02-20

**Authors:** Hirotaka Fukasawa, Ryuichi Furuya, Mai Kaneko, Daisuke Nakagami, Yuri Ishino, Shuhei Kitamoto, Kyosuke Omata, Hideo Yasuda

**Affiliations:** 1 Renal Division, Department of Internal Medicine, Iwata City Hospital, Iwata 438-8550, Shizuoka, Japan; 2 First Department of Medicine, Hamamatsu University School of Medicine, Hamamatsu 431-3192, Shizuoka, Japan

**Keywords:** anemia, cardiovascular disease, chronic kidney disease, copper, nutrition, zinc

## Abstract

The trace element zinc is essential for diverse physiological processes in humans. Zinc deficiency can impair growth, skin reproduction, immune function, maintenance of taste, glucose metabolism, and neurological function. Patients with chronic kidney disease (CKD) are susceptible to zinc deficiency, which is associated with erythropoiesis-stimulating agent (ESA) hypo-responsive anemia, nutritional problems, and cardiovascular diseases as well as non-specific symptoms such as dermatitis, prolonged wound healing, taste disturbance, appetite loss, or cognitive decline. Thus, zinc supplementation may be useful for the treatment of its deficiency, although it often causes copper deficiency, which is characterized by several severe disorders including cytopenia and myelopathy. In this review article, we mainly discuss the significant roles of zinc and the association between zinc deficiency and the pathogenesis of complications in patients with CKD.

## 1. Introduction

Zinc (Zn^2+^) is an essential trace element and the second most abundant divalent cation in the body next to iron (1.5–2.5 g in human body) [1]. Zinc plays an important role as a cofactor of more than 300 enzymes including alcohol dehydrogenase, alkaline phosphatase (ALP), angiotensin converting enzyme, carbonic anhydrase, collagenase, lactate dehydrogenase (LDH), and DNA and RNA polymerases (Table 1). Therefore, zinc is involved in the regulation of alcohol metabolism, bone metabolism, blood pressure control, cellular energy production, and nucleic acid synthesis [2,3,4,5]. Zinc also plays significant roles in the regulation of immune functions, genital functions, glucose metabolism, cognitive performance, and the structural maintenance of proteins, which are called zinc finger proteins including tumor necrosis factor (TNF)-α-induced protein 3 (TNFAIP3, also known as A20), nuclear factor-κB (NF-κB), nuclear factor erythroid 2-related factor 2 (Nrf2), and peroxisome proliferator-activated receptors (PPARs) [4,6,7,8]. In addition, zinc is essential in the active site of superoxide dismutase (SOD), an important antioxidant enzyme that catalyzes the dismutation of superoxide (O^−^) [9,10]. Thus, zinc acts as an antioxidant agent and zinc deficiency is associated with an increased risk of cardiovascular disease [11,12].

On the other hand, zinc deficiency is characterized by non-specific symptoms including weight loss, growth retardation, alopecia, dermatitis, prolonged wound healing, taste disturbance, appetite loss, and cognitive decline [13,14]. Therefore, zinc deficiency is often overlooked.

According to the recommended dietary zinc intakes from practical guidelines, the ideal daily dose for adults is 8 mg/day for women and 11 mg/day for men [15]. The dietary zinc content and its bioavailability can influence the efficiency of zinc absorption as well as an individual’s zinc status. Dietary zinc is actively absorbed throughout the small intestine; the main dietary sources of zinc include seafood (especially oysters), crustaceans, red meat, and poultry, although zinc’s bioavailability is lower in beans, nuts, and vegetables due to the presence of phytates [1]. Therefore, vegetarian or vegan diets may be a risk of zinc deficiency, especially in CKD patients. In the human body, 60% of zinc is stored in skeletal muscle and 20% in bones, while the circulating zinc accounts for only 0.1% of total body zinc [16]. In circulation, 80% of zinc is distributed in erythrocytes and 20% in serum, which is predominantly bound to several proteins such as albumin, α-macroglobulin, and transferrin [17]. In healthy populations, the major route of zinc excretion is via the gastrointestinal tract [18], although urinary excretion of zinc increases in patients with chronic kidney disease (CKD) [19].

In this review article, we mainly discuss the clinical significance of zinc and the association between zinc deficiency and the pathogenesis of complications in patients with CKD.

## 2. Zinc Levels in CKD

Zinc deficiency can be caused by nutritional problems and, therefore, it is very common in developing countries, mainly in children and the elderly. On the other hand, it can be complicated with chronic diseases such as diabetes mellitus, inflammatory bowel disease, CKD, or cancer [20].

Several studies have demonstrated that plasma zinc levels were lower in non-dialysis dependent CKD patients than those of healthy individuals and these levels decreased along the progression of CKD stages [19,21,22].

In patients undergoing hemodialysis (HD) treatment, previous studies have demonstrated that circulating zinc levels were lower than those of healthy individuals [23,24]. Toida et al. [25] have also reported that serum zinc levels in most of incident hemodialysis patients (99.2%) were under the normal range (serum zinc level < 80 mg/dL) and 70.4% patients exhibited hypozincemia (serum zinc level < 60 mg/dL).

In patients undergoing peritoneal dialysis (PD) treatment, Panorchan et al. [26] have reported that mean plasma zinc levels were relatively low, with 57.2% of the patients under the normal range. Recently, Shimizu et al. [27] have reported that serum zinc levels in all PD patients (n = 47) were under the normal range and that there was no significant difference in the prevalence of zinc deficiency between PD and HD patients.

**Figure 1:**
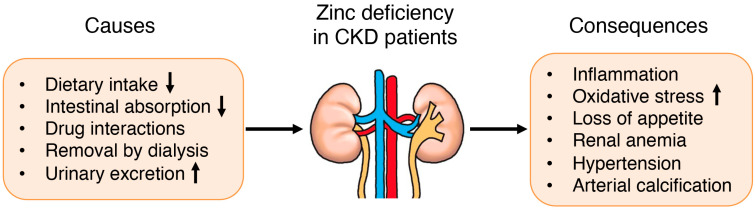
Causes and consequences of zinc deficiency in patients with CKD.

Thus, CKD patients are susceptible to zinc deficiency, which may be caused by an inadequate dietary intake due to uremia-related anorexia and dietary restriction, reduced gastrointestinal zinc absorption, adsorption of zinc by phosphate binders, and removal of zinc by dialysis procedure, which usually uses zinc-free dialysate (Figure 1) [24,28]. In addition, it is possible that CKD patients have variable susceptibility to zinc deficiency on the basis of several factors including genetic variation in the zinc transporter genes and relevant transcription factors, long-term diuretic use, and the original disease of CKD such as diabetes mellitus. However, Batista et al. [29] have reported that there was no significant difference in plasma zinc levels in hemodialysis patients with or without diabetes mellitus.

On the other hand, previous studies have demonstrated that zinc levels in erythrocytes were higher in non-dialysis dependent CKD patients than those in healthy individuals, while zinc levels in plasma were lower in the aforementioned patients [29,30]. These results suggest that zinc in circulation is differently distributed between CKD patients and healthy individuals.

## 3. Zinc and Renal Anemia

Renal anemia is a common complication in patients with CKD [31,32]. Until quite recently, the main therapeutic options for renal anemia were treatment with an erythropoiesis-stimulating agent (ESA) and iron supplementation. On the other hand, a problem in treating renal anemia is that the ESA dosage required to achieve the target hemoglobin level widely varies among CKD patients, so called as ESA hypo-responsiveness. Although several factors were reported to contribute to ESA hypo-responsiveness, including iron deficiency, inflammation, infection, inadequate dialysis procedure, and severe hyperparathyroidism [33,34], recent studies have demonstrated that zinc deficiency could also cause ESA hypo-responsiveness, particularly in patients undergoing HD [35,36].

In fact, Fukushima et al. [35] have showed that serum zinc levels were positively correlated with anemic parameters such as red blood cell (RBC) counts, hemoglobin (Hb), or hematocrit (Ht) levels in HD patients with lower zinc levels than the reference value (<80 mg/dL), and that zinc supplementation with polaprezinc (as 34 mg/day of zinc) could improve anemia and reduce ESA doses in those patients. Kobayashi et al. [36] have also showed that zinc supplementation with polaprezinc reduced serum ferritin levels, required ESA dosage, and erythropoietin responsiveness index, although it didn’t change anemic parameters (RBC and Hb) in HD patients.

Although no previous studies have directly shown a relationship between zinc levels and Hb production or erythropoiesis, some experimental studies have reported that zinc finger proteins including BTB and CNC homology-1 (Bach-1), GATA-1, and growth factor independence-1B (Gfi-1B) play important roles in Hb synthesis and erythroid proliferation or differentiation [37,38,39]. Therefore, it is speculated that the improvement of renal anemia following zinc supplementation is caused by Hb synthesis and erythropoiesis via the functional modification of those transcription factors containing zinc, although the precise mechanism for how zinc deficiency affects those transcription factors in vivo remains unclear. Further studies are needed to clarify its mechanism.

## 4. Zinc and Nutrition in CKD

CKD patients are often suffering from nutritional problems, which are associated with increased morbidity and mortality [40]. In fact, body mass index (BMI [reference range; 18.5≤ to <25.0]) in CKD patients exhibits lower than age- and sex-matched control subjects [41]. Several studies have demonstrated that higher BMI contributed to a survival advantage in CKD patients [42,43]. Since higher BMI is related to an increased risk of cardiovascular diseases and a higher mortality in the general population [44], this reverse relationship observed in CKD patients is known as the “risk factor paradox” or “reverse epidemiology” [45,46]. On the other hand, it is unclear whether this survival advantage associated with higher BMI in CKD patients is caused by increased muscle mass, fat mass, or both. One possible reason for why this question remains unclear is because BMI does not distinguish between muscle mass and adipose tissue [43]. In this regard, Beddhu et al. [47] have attempted to answer this question using 24-h urinary creatinine excretion as a marker for muscle mass in conjunction with BMI and proposed that muscle mass might be more important in this survival advantage than fat mass. Besides, Caetano et al. [48] have demonstrated that fat mass might be more important than muscle mass in predicting 1-year mortality with bioimpedance analysis.

Previously, El-Shazly et al. [49] have reported that serum zinc levels were positively correlated with body weights and BMIs, but negatively correlated with serum leptin levels in pediatric patients on dialysis. Several studies have also demonstrated that zinc supplementation resulted in a significant increase in body weights and BMIs, but a significant decrease in serum leptin levels in HD patients [49,50]. Therefore, it is suggested that zinc levels are associated with body composition in CKD patients, at least partially, although it remains uncertain whether muscle mass or fat mass was increased by zinc supplementation.

Recently, we have reported that serum zinc levels were positively correlated with the abdominal fat areas of HD patients [51]. In the experimental study, it has been reported that zinc stimulated the differentiation of pre-adipocytes to adipocytes in vitro [52]. Another report has demonstrated that zinc supplementation caused the increased size of adipocytes resulting in the adipose tissue hypertrophy in mice [53]. Zhang et al. [54] have reported that dietary zinc supplementation increased intramuscular adipose deposition in piglets. Chen et al. [55] have also reported that zinc supplementation for 6 weeks caused fat accumulation in the body of genetically obese mice and dietary-obese mice. These reports support the idea that zinc mainly affects adipose tissue in CKD patients, although further studies are needed to clarify the mechanism for how circulating zinc levels affect body composition.

## 5. Zinc and Cardiovascular Diseases in CKD

At present, it has become clearer that zinc deficiency is associated with oxidative stress, inflammation, and the development of cardiovascular diseases in CKD patients [12].

Lobo et al. [53,54] have reported that plasma zinc levels were negatively correlated with electronegative low-density lipoprotein [LDL(-), a lipid peroxidation and pro-atherosclerotic marker] and TNF-α levels in hemodialysis patients and have proposed that zinc deficiency may cause oxidative stress, inflammation, and subsequently, atherosclerosis.

Vascular calcification is a common complication in CKD patients and is a significant predictor of cardiovascular mortality [55]. Several studies have demonstrated that abdominal aortic calcification is significantly associated with cardiovascular events in CKD patients [56,57]. The pathophysiology of vascular calcification in CKD patients involves several factors including oxidative stress, inflammation, changes in extracellular matrix metabolism, and imbalances in calcium-phosphate metabolism referred to as CKD-mineral and bone disorder (CKD-MBD) [58,59]. Voelkl et al. [6] have reported that serum zinc levels were negatively correlated with a propensity for serum calcification in CKD patients and that zinc sulfate supplementation suppressed vascular calcification in CKD model mice via the increased aortic expression of TNFAIP3, which is a suppressor of the NF-κB transcription factor pathway. Zinc deficiency also activated the NACHT, LRR, and PYD domains-containing protein 3 (NLRP3) inflammasome and induced interleukin-1β (IL-1β) secretion in an animal model of acute kidney injury [60], although zinc treatment inhibited the activation of the NLRP3 inflammasome by the attenuation of reactive oxygen species (ROS) production in human peritoneal mesothelial cells [61].

Nrf2 is a transcription factor that regulates the cellular defense against oxidative stress by reducing ROS overproduction. Nrf2 also blocks inflammation by directly inhibiting transcription of the proinflammatory cytokine genes or inhibiting the activity of NF-κB signaling [8,62]. Previous study has demonstrated that CKD patients exhibited both downregulation of Nrf2 mRNA and upregulation of NF-κB mRNA expression, and that zinc supplementation caused increased Nrf2 expression as well as enhanced SOD synthesis, improved antioxidant defense, and reduced cardiovascular risk in CKD patients [63].

Systematic review and meta-analysis have reported the benefits of zinc supplementation on oxidative stress and inflammation, which resulted from the increase in SOD levels and the decrease in malonaldehyde and C-reactive protein (CRP) levels [64].

## 6. Zinc Supplementation and Risk of Copper Deficiency in CKD

Besides zinc, copper is also an essential trace element in physiological processes such as the regulation of oxidative stress, catecholamine metabolism, or hematopoiesis [22,65], although zinc supplementation can induce acquired copper deficiency known as zinc-induced copper deficiency (ZICD) [66]. ZICD can induce severe disorders including ESA hypo-responsive anemia, pseudo-myelodysplastic syndrome, or myelopathy [67,68,69], and several cases of ZICD have been reported in hemodialysis patients [70,71,72]. On the other hand, ZICD is relatively uncommon and, therefore, is often overlooked as a cause of anemia, pancytopenia, or myelopathy in patients with CKD.

Absorption of both zinc and copper occurs in the small intestine and is dependent on the relative concentrations of each element. The pathophysiology for ZICD may be explained by the interaction of copper and zinc with metallothionein (MT) proteins in the enterocytes of the small intestine. MT proteins form disulfide bonds with metals such as cadmium, zinc, and copper, and help maintain stable metal ion levels in the body [73]. The increased zinc concentration stimulates an increased synthesis of MT proteins, which results in more binding sites for both copper and zinc on MT proteins. Since copper has a greater binding affinity to MT proteins than zinc and the turnover rate of enterocytes is relatively rapid, copper bound to MT proteins is unable to be absorbed in the small intestine and is finally lost in the stool. Thus, ZICD can occur in CKD patients if zinc levels are remarkably high after zinc supplementation [70,71,72].

## 7. Conclusions and Future Perspectives

CKD patients are susceptible to zinc deficiency, which may often cause ESA hypo-responsive anemia, nutritional problems, or cardiovascular diseases as well as non-specific symptoms including dermatitis, prolonged wound healing, taste disturbance, and appetite loss. Although zinc supplementation is a useful treatment for CKD patients with its deficiency, risk of ZICD should be noted. Further studies are needed to determine how to manage zinc deficiency in CKD patients.

## Figures and Tables

**Table 1:** Main enzymes containing zinc, existing organs, and their functions.

Enzyme	Existing Organs	Functions
Alkaline phosphatase (ALP)	liver, bone, placenta, small intestine	dephosphorylation, bone metabolism
Alcohol dehydrogenase	liver, stomach, intestinal tract, kidney	alcohol metabolism
Aldolase	muscle, liver	glucose metabolism
Alkaline protease	small intestine	protein metabolism
Amylase	salivary gland, pancreas, small intestine	protein metabolism
Angiotensin coverting enzyme	lung, kidney, brain	regulation of blood pressure
Carbonic anhydrase	red blood cell	exchange between carbon dioxide and bicarbonate ion
Carboxypeptidase	pancreas, liver, kidney, small intestine	protein metabolism
Collagenase	all organs	hydrolysis of collagen
Dipeptidase	small intestine	protein metabolism
DNA polymerase	all organs	DNA synthesis
Glutamate dehydrogenase	liver	protein metabolism
Lactate dehydrogenase (LDH)	most organs	glucose metabolism
Leucine aminopeptidase	liver, kidney, intestinal tract, pancreas	protein metabolism
Ornithine transcarbamylase	liver	protein metabolism, nitrogen metabolism
Phospholipase C	all organs	lipid metabolism
RNA polymerase	all organs	RNA synthesis
Superoxide dismutase (SOD)	all organs	anti-oxidative stress, reactive oxygen suppression

Abbreviations: ALP, alkaline phosphatase; DNA, deoxyribonucleic acid; LDH, lactate dehydrogenase; RNA, ribonucleic acid; SOD, superoxide dismutase.

## Data Availability

Not applicable.
